# A survey of resilience, burnout, and tolerance of uncertainty in Australian general practice registrars

**DOI:** 10.1186/1472-6920-13-2

**Published:** 2013-01-07

**Authors:** Georga PE Cooke, Jenny A Doust, Michael C Steele

**Affiliations:** 1 Centre for Research in Evidence-Based Practice, Bond University, Gold Coast, Queensland, Australia; 2 Faculty of Business, Bond University, Gold Coast, Queensland, Australia

**Keywords:** Adaptation, Psychological, Burnout, Professional, Job satisfaction, Uncertainty

## Abstract

**Background:**

Burnout and intolerance of uncertainty have been linked to low job satisfaction and lower quality patient care. While resilience is related to these concepts, no study has examined these three concepts in a cohort of doctors. The objective of this study was to measure resilience, burnout, compassion satisfaction, personal meaning in patient care and intolerance of uncertainty in Australian general practice (GP) registrars.

**Methods:**

We conducted a paper-based cross-sectional survey of GP registrars in Australia from June to July 2010, recruited from a newsletter item or registrar education events. Survey measures included the Resilience Scale-14, a single-item scale for burnout, Professional Quality of Life (ProQOL) scale, Personal Meaning in Patient Care scale, Intolerance of Uncertainty-12 scale, and Physician Response to Uncertainty scale.

**Results:**

128 GP registrars responded (response rate 90%). Fourteen percent of registrars were found to be at risk of burnout using the single-item scale for burnout, but none met the criteria for burnout using the ProQOL scale. Secondary traumatic stress, general intolerance of uncertainty, anxiety due to clinical uncertainty and reluctance to disclose uncertainty to patients were associated with being at higher risk of burnout, but sex, age, practice location, training duration, years since graduation, and reluctance to disclose uncertainty to physicians were not.

Only ten percent of registrars had high resilience scores. Resilience was positively associated with compassion satisfaction and personal meaning in patient care. Resilience was negatively associated with burnout, secondary traumatic stress, inhibitory anxiety, general intolerance to uncertainty, concern about bad outcomes and reluctance to disclose uncertainty to patients.

**Conclusions:**

GP registrars in this survey showed a lower level of burnout than in other recent surveys of the broader junior doctor population in both Australia and overseas. Resilience was also lower than might be expected of a satisfied and professionally successful cohort.

## Background

Doctors are widely reported to be unhappy and burnt-out [1]. Burnout is a psychological “syndrome of emotional exhaustion, depersonalization, and reduced personal accomplishment that can occur among individuals who work with people” [2]. The onset of burnout is often in the early postgraduate period [3]. Willcock found that the prevalence of burnout increased steadily through the first year of a medical practitioner’s career [4].

A doctor’s affective reactions to clinical uncertainty, bad patient outcomes and coping behaviours, as measured by the Physician Response to Uncertainty scale, impacts on how they respond to stress [5]. Higher intolerance of clinical uncertainty has been linked to burnout in one study of primary care physicians [6] and one study of emergency physicians [7] in the USA. General practitioners and other primary care practitioners who see a high proportion of undifferentiated illness are particularly exposed to uncertainty in decision making. Doctors with higher anxiety about uncertainty tend to have higher costs of investigation and treatment [8].

With the rise of positive psychology, resilience and positive adaptation have become a field of research in themselves. Resilience has various definitions but is broadly accepted to mean a “dynamic, evolving process of positive attitudes and effective strategies” that we employ in response to life stressors [9]. The value of resilience is widely acknowledged in nursing literature [10] but it has attracted much less attention in medical circles. One study demonstrated that resilience is associated with academic productivity in minority faculty members in a US academic health centre [11]. Keeton used an unvalidated scale of resilience to examine predictors of career satisfaction in a population of US physicians [12]. Recently, the concept of vicarious resilience, which acknowledges the “complex potential of therapeutic work both to fatigue and to heal” [13] has come to the attention of psychotherapists. Junior doctors are similarly exposed to caring for people who appear to thrive in life despite ill health and suffering. However, vicarious resilience in doctors has not yet been the focus of research activity.

To summarise, early in their career, junior doctors must face being held accountable for their medical decisions for the first time. They must learn to manage uncertainty, but it is also when they are vulnerable to burnout. We hypothesised that burnout, resilience and tolerance of uncertainty are likely to be related in junior doctors. However, we were unable to identify a study which explored if a relationship existed between these three concepts. The aim of this study was to measure the prevalence of resilience, burnout, compassion satisfaction and tolerance of uncertainty in Australian GP registrars, to determine if there are relationships between these concepts in this practice group. We also aimed to determine if demographic factors and personal meaning derived from patient care were associated with burnout in this group.

## Methods

We conducted a paper-based survey of Australian GP registrars. This study had ethics approval from the Bond University Human Research Ethics Committee.

### Participants

Australian GP registrars were eligible for participation if they had completed at least 2 years of hospital-based posts and at least three months in GP rotations. In Australia, GP registrars are doctors on a formal training program to become independently registered general practitioners, known as family doctors in some other parts of the world. Before commencing the program, GP registrars must have completed at least one-year of a hospital based internship. Once accepted on the program, the most common pathway involves three years of supervised training: one year of hospital training, 18 months within community general practice and 6 months of extended skills training. On completion of training time and other requirements, including a fellowship examination, registrars become qualified to practise independently as general practitioners. Extended pathways exist for registrars wishing to gain advanced skills for rural general practice.

### Recruitment

We used a convenience sample and recruited participants by advertising in a GP registrar newsletter and by approaching registrars at education events put on by four Regional Training Providers (RTPs) in Queensland, Victoria, South Australia and the Northern Territory who deliver GP registrar training in Australia. With the permission of the Directors of Education at each RTP, the primary researcher attended education events to distribute the survey. The recruitment period was June 2010.

### Setting and procedure

Participants recruited at education events could complete the survey at the event or return it via the mail at a later date. We distributed the survey with a reply-paid envelope, together with an introductory letter and explanatory statement. We did not offer incentives or inducements.

### Survey instruments

Our survey consisted of several validated scales, including for burnout, resilience, tolerance of uncertainty and personal meaning in patient care (Table 1). We also included questions about demographics and progress through GP training.

**Table 1:** Instruments included in survey

**Name of instrument**	**Range of possible scores**
**Single item measure of burnout**	1 to 5
**Professional Quality of Life (ProQOL)**	
Compassion satisfaction (CS)	10 to 50
Burnout	10 to 50
Secondary Traumatic Stress (STS)	10 to 50
**Resilience Scale - 14**	14 to 98
**Personal Meaning in Patient Care**	6 to 24
**Intolerance to Uncertainty Scale – 12**	
Prospective anxiety	7 to 35
Inhibitory anxiety	5 to 25
Total	12 to 60
**Physician Reactions to Uncertainty**	
Anxiety due to uncertainty	5 to 30
Concern about bad outcomes	3 to 18
Reluctance to disclose uncertainty to patients	5 to 30
Reluctance to disclose uncertainty to physicians	2 to 12

The primary measure of burnout was the single-item measure of burnout [14-16] which has been validated against the Maslach Burnout Inventory emotional exhaustion subscale, a widely used scale for burnout, in a cohort of physicians [17] and Australian clinical cancer workers [18]. This measure consists of five statements (Table 2), with participants asked to select the most relevant statement for them. Participants were divided into ‘low risk of burnout’ if they selected statement 1 or 2, and ‘high risk’ if they selected statements 3-5, as described by Rohland [17]. The Professional Quality of Life (ProQOL) scale was also used to measure burnout, together with secondary traumatic stress and compassion satisfaction [19]. The ProQOL subscale for burnout has 10 items. The ProQOL subscale is divided into low, average and high scores for burnout according to predefined cut-offs.

**Table 2:** **Statements in single item measure of burnout**[14-16]

**Response number**	***Statement***
1	I enjoy my work. I have no symptoms of burnout.
2	Occasionally I am under stress, and I don’t always have as much energy as I once did, but I don’t feel burned out.
3	I am definitely burning out and have one or more symptoms of burnout, such as physical and emotional exhaustion.
4	The symptoms of burnout that I’m experiencing won’t go away. I think about frustration at work a lot.
5	I feel completely burned out and often wonder if I can go on. I am at the point where I may need some changes or may need to seek some sort of help.

The Resilience Scale-14 measures global resilience, comprised of five characteristics: purpose, perseverance, self-reliance, equanimity and existential aloneness [20]. The 14 items are scored on a likert scale, which are summed to arrive at a global score. The authors divide the global score into three categories. For simplicity, we refer to these as low (L), low-moderate (M), and high (H) resilience. The Personal Meaning in Patient Care scale, consisting of six items, was validated in clinicians working in genetics [21]. High scores are associated with low burnout, gratitude and professional satisfaction.

We measured intolerance of uncertainty both generally and in the clinical context by using two separate scales. The Intolerance of Uncertainty-12 (IUS-12) scale has twelve items each scored on a likert scale of 5. The IUS-12 can be divided into two subscales; one for *prospective* anxiety (fear and anxiety based on future events) (seven items) and one for *inhibitory* anxiety (uncertainty which inhibits action or experience) (five items) [22]. The Physician Reactions to Uncertainty scale measures affective reactions to uncertainty in clinical situations, using four subscales which are summarised in Table 1[5].

### Analysis

Participants were categorised as being at high or low risk of burnout using the single-item measure of burnout. Relationships between burnout and non-burnout groups were determined using t-tests for continuous data and χ^2^ tests for categorical data. For the resilience analysis, where low, low-moderate, and high resilience could not be assumed to be normally distributed, a Kruskal-Wallis test was used instead of an ANOVA. Cronbach’s α was used to measure reliability. All analyses were conducted with SPSS software.

## Results

We distributed 148 surveys and received 128 responses (90% response rate). 122 surveys were distributed at education events (98%). The demographic and geographic distribution of respondents (Table 3) was similar to the Australian GP registrar cohort [23]. Most registrars felt that their previous experience had prepared them well for general practice (80%) and felt well supervised in their training (89%).

**Table 3:** Participant Characteristics

**Participant characteristics**	**%**
**Sex**	
Female	67
**Age**	
20 – 29	44
30 – 39	41
40 – 49	11
50+	4
**Years since graduation**	
3	17
4	28
5	16
6 to 10	25
11+	15
**GP Training Term**	
Term 1 (0-6 months full time equivalent)	66
Term 2 (7-12 months full time equivalent)	6
Term 3 (13-18 months full time equivalent)	27
Other	1
**Practice Location**	
Metropolitan	64

Eighteen registrars (14%) were at risk of burnout when using the single-item burnout scale. Using the ProQOL burnout subscale, no registrars had scores suggesting a high risk of burnout, although those who were at high risk of burnout on the single-item scale also had statistically significantly higher scores for burnout on the ProQOL (p <0.001). The internal reliability of the ProQOL, Intolerance of Uncertainty-12 scale and Physician Response to Uncertainty scale were good to excellent (Cronbach’s α >0.75) and were consistent with the results in validation studies.

Sex, age, practice location and time in general practice showed no relationship with burnout (Table 4). Burnout was associated with secondary traumatic stress but not with compassion satisfaction. Lower resilience, lower personal meaning in patient care, intolerance of uncertainty, prospective anxiety, inhibitory anxiety, anxiety due to (clinical) uncertainty and reluctance to disclose uncertainty to patients were associated with burnout (Table 5).

**Table 4:** Relationship between demographic factors and risk of burnout

**Participant characteristic**	***χ*2 test of independence (p value)**
Sex	0.96
Age	0.80
Years since graduation	0.74
GP Term	0.65
Practice Location (rural or metropolitan)	0.19

**Table 5:** The relationship of burnout to compassion satisfaction, secondary traumatic stress, personal meaning in patient care and intolerance of uncertainty in Australian GP registrars

	**Low burnout (n = 109) Mean (SD)**	**High burnout (n = 18) Mean (SD)**	**p value**
**ProQOL**			
Compassion satisfaction	36.8 (4.4)	33.9 (5.9)	0.63
Secondary traumatic stress	20.8 (4.0)	24.2 (4.6)	0.002*
**Intolerance of Uncertainty Scale** -**12**			
Prospective anxiety	19.7 (4.6)	23.3 (6.5)	0.005 *
Inhibitory anxiety	10.5 (3.8)	13.3 (4.3)	0.005 *
Total	30.2 (7.2)	36.6 (9.8)	0.001 *
**Resilience Scale -14**	75.8 (12.5)	67.7 (10.0)	0.010 *
**Personal meaning in patient care**	16.8 (3.2)	14.7 (3.3)	0.014 *
**Physician response to uncertainty scale**			
Anxiety due to uncertainty	18.8 (5.0)	21.7 (4.3)	0.02 *
Concern about bad outcomes	11.5 (3.4)	13.0 (4.0)	0.10
Reluctance to disclose uncertainty to patients	13.6 (3.8)	15.7 (3.3)	0.03*
Reluctance to disclose uncertainty to physicians	4.0 (1.9)	4.5 (2.1)	0.30

*statistically significant result.

Most registrars had scores which fell in the low-moderate resilience range (82%). Only ten registrars (8%) had high resilience scores and thirteen registrars (10%) had a low resilience score. High resilience was associated with low burnout (P <0.001) and low general intolerance to uncertainty (P = 0.049). In relation to clinical uncertainty, resilience was associated with less concern about bad outcomes (P = 0.001) and less reluctance to disclose uncertainty to patients (P = 0.007). However, no relationship was found between reluctance to disclose uncertainty to other physicians (P = 0.1) and with anxiety due to uncertainty (P = 0.5).

## Discussion

Our survey found a lower prevalence of burnout in GP registrars (14% using the single-item scale and none using the ProQOL burnout subscale) than other recent surveys of the Australian junior doctor population (31 – 75%) [4,24,25]. Interestingly, we found no relationship between a registrar’s duration of GP experience and burnout. While negative responses to clinical uncertainty have been linked with burnout in other research [6,7], the relationship between lower resilience and lower tolerance of clinical uncertainty has not been demonstrated before. We are also not aware that a doctor’s general response to uncertainty (IUS-12) has been linked with burnout or resilience previously. We found no relationship between burnout or resilience and reluctance to disclose uncertainty to physicians (p = 0.30); a reassuring finding as GP registrars will generally need to seek help when they encounter unfamiliar situations.

Our study had several strengths. We achieved a very high response rate and our sample was representative of the GP registrar population when compared with the most comprehensive data available [23]. The limitations of our study are common to most burnout literature. While the single-item questionnaire has been validated in a cohort of physicians, it may not be sufficiently reliable. However, our second measure of burnout, the 10 item ProQOL scale, is likely to be sensitive and has been widely used in previous research and showed a similar direction of results. Also, the surveys were distributed at educational events, where registrars may feel more positive towards their work compared to a day of usual clinical practice.

Low response rates and responder bias may explain the higher level of burnout (31 – 75%) reported in other studies [4,24,25]. Our results are surprising but are in line with the validation study of the single-item measure of burnout in US physicians, which found that 23% of physicians were burnt-out [17], and with a recent survey of GP registrars that showed 10% were mildly or moderately depressed or anxious [26]. GP registrars may be less burnt-out than their hospital colleagues during training, or our results may represent a broader trend where junior doctors are not as burnt-out as their more senior colleagues.

Burnout has significant consequences for a doctor’s own health and that of their patients. For example, internal medicine trainees who are burnt-out are more likely to self-report suboptimal patient care [27] and medical errors [8]. Burnout is also linked with the intention to leave clinical medicine [14]. Montgomery [28] has proposed that burnout is the missing link in quality care. Indeed, burnout and physician wellness have been described as neglected quality indicators in medicine [29].

Our study showed burnout in GP registrars is strongly linked with general intolerance of uncertainty. GP training in Australia provides a structured opportunity to intervene to prevent burnout and bolster tolerance of uncertainty. Discussing tolerance of uncertainty and evidence-based management of clinical uncertainty may be a more acceptable approach for registrars, rather than overtly talking about burnout prevention.

Resilience was linked to high compassion satisfaction, low burnout, and a higher tolerance of both general and clinical uncertainty. However, resilience was also lower than might be expected. While it is increasingly acknowledged that teaching and learning about resilience is important in health professional curricula [30], there is scant research about resilience and medical professionals. Jensen identified factors which contribute to physician resilience which can be learnt by physicians (accepting personal limitations, developing self-awareness and limit setting) or addressed structurally within a workplace (practice management style and culture) [9]. A longitudinal study of GP registrars, with adequate response rates, through fellowship and beyond, would be required to explain our findings.

## Conclusions

Overall, our study showed a lower prevalence of burnout than would be expected from recent Australian data. Burnout in GP registrars is also strongly linked with general intolerance of uncertainty. Resilience was also lower than might be expected. Resilience was linked to high compassion satisfaction, low burnout, and a higher tolerance of both general and clinical uncertainty.

## Competing interests

Georga Cooke is the immediate past Registrar Representative on the Royal Australian College of General Practitioners Council. She was employed by General Practice Education and Training (GPET) Ltd as the Registrar Research and Development Officer in 2011, a registrar position which assists with coordinating academic registrar posts in GP training in Australia, and now sits on the GPET Board.

## Authors’ contributions

GPEC was chiefly responsible for the conception, design and coordination of the study and analysis, and drafted the manuscript. JAD and MCS participated in the design of the study and helped to draft the manuscript. MCS performed the statistical analysis. All authors have read and approved the final manuscript.

## Pre-publication history

The pre-publication history for this paper can be accessed here:

http://www.biomedcentral.com/1472-6920/13/2/prepub
